# RELATION OF PREFRONTAL CORTEX FUNCTION WITH DUAL-TASK GAIT DEPENDS ON POSTDORSAL CAUDATE DOPAMINE

**DOI:** 10.1093/geroni/igae098.1057

**Published:** 2024-12-31

**Authors:** Andrea Rosso, Emma Baillargeon, Theodore Huppert, Nicholaas Bohnen, Lana Chahine, Caterina Rosano

**Affiliations:** University of Pittsburgh, Pittsburgh, Pennsylvania, United States; University of Pittsburgh, Pittsburgh, Pennsylvania, United States; University of Pittsburgh, Pittsburgh, Pennsylvania, United States; University of Michigan, Ann Arbor, Michigan, United States; University of Pittsburgh, Pittsburgh, Pennsylvania, United States; University of Pittsburgh, Pittsburgh, Pennsylvania, United States

## Abstract

In older adults, the extent of PFC activation during challenged walking may be indicative of either compensation (if performance is maintained) or of neural inefficiencies (if performance declines). Dopamine neurotransmission in the basal ganglia is a determinant of motor automaticity and could therefore be a driver of compensatory vs inefficient PFC activity. We tested whether associations of change in PFC activation measured by functional near-infrared spectroscopy with change in gait speed from single- to dual-task (alternate letter of the alphabet) walking over 15 meters differed by dopamine binding in a cohort of older adults (n=87, age=73±5 years, 67% women, gait speed=1.06±0.81 m/s). Dopamine neurotransmission was measured by dihydrotetrabenazine ([11C]DTBZ) positron emission tomography in the postcommissural caudate, which is highly connected to the dorsolateral PFC. Linear regression analyses assessed multiplicative interactions between PFC activation and [11C]DTBZ binding (above/below median) adjusted for age and gender with gait speed change as the dependent variable. Greater PFC activation was non-significantly related to greater decline in gait speed during dual-task. There was a significant interaction (p=0.05) between [11C]DTBZ in the postcommissural caudate and PFC function such that for those with higher dopamine binding, there was no association between PFC activation and gait speed change (beta=-0.002, p=0.8) but in those with lower dopamine, greater increase in PFC activation was associated with greater decline in gait speed from single- to dual-task walking (beta=-0.03, p=0.02). Higher PFC activation among those with lower dopamine binding may be indicative of neural inefficiencies that result in poor dual-task performance.

